# Transgenic Epigenetics: Using Transgenic Organisms to Examine Epigenetic Phenomena

**DOI:** 10.1155/2012/689819

**Published:** 2012-03-27

**Authors:** Lori A. McEachern

**Affiliations:** ^1^Department of Biology, Dalhousie University, Halifax, NS, Canada B3H 4R2; ^2^Department of Physiology, Dalhousie University, Halifax, NS, Canada B3H 4R2

## Abstract

Non-model organisms are generally more difficult and/or time consuming to work with than model organisms. In addition, epigenetic analysis of model organisms is facilitated by well-established protocols, and commercially-available reagents and kits that may not be available for, or previously tested on, non-model organisms. Given the evolutionary conservation and widespread nature of many epigenetic mechanisms, a powerful method to analyze epigenetic phenomena from non-model organisms would be to use transgenic model organisms containing an epigenetic region of interest from the non-model. Interestingly, while transgenic *Drosophila* and mice have provided significant insight into the molecular mechanisms and evolutionary conservation of the epigenetic processes that target epigenetic control regions in other model organisms, this method has so far been under-exploited for non-model organism epigenetic analysis. This paper details several experiments that have examined the epigenetic processes of genomic imprinting and paramutation, by transferring an epigenetic control region from one model organism to another. These cross-species experiments demonstrate that valuable insight into both the molecular mechanisms and evolutionary conservation of epigenetic processes may be obtained via transgenic experiments, which can then be used to guide further investigations and experiments in the species of interest.

## 1. Introduction

Transgenic model organisms have been widely used to study a variety of epigenetic processes and mechanisms. The majority of these studies have examined epigenetic control regions (i.e., DNA sequences targeted by epigenetic modifications, also referred to herein as epigenetic sequences) that have been relocated to a novel chromosomal position in the same model organism, an approach that can provide valuable information regarding the minimum sequences required at the endogenous locus, as well as the mechanisms and proteins that contribute to epigenetic expression or repression [1–6]. An alternative, but less used, type of transgenic epigenetic study involves transferring an epigenetic control region from one species into another. This cross-species approach can provide valuable insight into the molecular mechanisms that act on an epigenetic sequence of interest, which may be difficult to study at the endogenous locus, and can be facilitated in transgenic studies by including easy-to-monitor reporter genes adjacent to the epigenetic sequence in the transgenic construct. In addition, this method holds tremendous potential in the study of the evolution of epigenetic mechanisms, allowing for the rapid determination of whether an epigenetic process is based on widespread, evolutionary conserved mechanisms that are found in a wide range or eukaryotes, or whether it is a species-specific unique process.

Despite the great potential of this technique, it has thus far been vastly underutilized and has not yet been employed in the study of non-model organism epigenetics. Non-model organisms are traditionally difficult to work with in a laboratory environment for a wide range of reasons, including size, life cycle, viability, breeding ability, and a lack of well-established propagation- and housing-methods. In addition, non-model organisms generally lack genetic and epigenetic tools and protocols that are well developed, widely tested, and accepted within the scientific community. By transferring an epigenetic sequence of interest from a non-model organism to an amenable model organism for which a plethora of tools are available, such as *Drosophila* or mice, new information regarding how the original sequence works may be obtained. For example, this method can be used to identify the minimum sequence required for epigenetic effect on gene expression, the identity of DNA regulatory elements contained within the sequence, the presence or absence of methylation at the sequence, and whether the sequence stimulates the formation of a compact heterochromatin domain. Furthermore, analysis of proteins and protein complexes bound to the sequence, histone modifications acquired by the sequence, the effect of small interfering RNA (siRNA) or short hairpin RNA (shRNA) knockdowns, and the effect of DNA methylation- or histone modification-inhibitors may be more quickly and easily examined in a transgenic model organism than in the original non-model organism. Finally, the transgenic approach may be especially useful to quickly and thoroughly examine the effect of a wide range of mutant strains or genetic knockouts on the epigenetic sequence of interest, as well as the inheritance pattern of the epigenetic state across several generations.

This cross-species transgenic approach is predicated on the assumption that epigenetic processes and proteins are evolutionary conserved, and that an epigenetic process can be studied in a transgenic environment. These assumptions will be examined here by detailing several cross-species transgenic epigenetic experiments that studied the processes of genomic imprinting and paramutation, by transferring epigenetic control sequences from one model organism to another.

## 2. Conserved Epigenetic Mechanisms

Epigenetic effect on gene expression is accomplished by a variety of molecular mechanisms that lead to gene expression or repression, including histone modifications, changes in higher-order chromatin structure, DNA methylation, RNA interference (RNAi), and noncoding RNAs. These mechanisms have been observed in a wide range of organisms, from yeast to plants and mammals, suggesting that they are both widespread and evolutionary conserved [7–13].

Histone modifications are at the very core of epigenetic gene regulation, and many other epigenetic processes ultimately contribute to the epigenetic status of a locus by directing or targeting modifications of histone proteins. DNA is packaged within the nucleus by its association with nucleosomes, protein structures that consist of two copies of four different histone proteins (H2A, H2B, H3, and H4). This complex of DNA and protein is termed chromatin; densely packed “inactive” chromatin is termed heterochromatin, while loosely packed chromatin is termed euchromatin. Chemical modifications of amino acids in the histone proteins, such as methylation, acetylation, phosphorylation, sumoylation, ubiquitination, and ribosylation, can lead to the formation of heterochromatin or euchromatin, depending on the nature and position of the modification. The inclusion of variant histones, and the availability of histone chaperones, can also contribute to changes in chromatin structure [14–16].

Further changes in higher-order chromatin structure may be facilitated by DNA-binding proteins that mediate the formation of chromatin loops or other complex chromatin structures and thereby modify the access of regulatory proteins, chromatin remodelling proteins, and histone modification enzymes, to their target sequences or sites. These DNA-binding proteins and higher-order chromatin structures may also contribute to epigenetic gene expression by localizing the target sequences to a particular region within the nucleus [17]. Maintenance of silent or active chromatin states often also involves the well-characterized Polycomb group (PcG) and trithorax group (trxG) proteins, which regulate the expression of many developmental genes and exhibit extensive evolutionary conservation in eukaryotes, with homologues identified in fungi, plants, and animals [18]. These proteins form large multimeric complexes that maintain transcriptional repression and activation, primarily by directing histone modifications and chromatin remodelling [18]. Epigenetic processes mediated by PcG and other chromatin proteins have also been observed to involve noncoding RNAs, small RNAs, and the RNAi pathway, demonstrating the interconnectedness of these epigenetic mechanisms [19–21]. Importantly, a number of studies have demonstrated the functional conservation of this family of proteins and other chromatin modifiers, by showing that *Drosophila* PcG proteins can function as repressors in mammalian cells [22], and mammalian homologues can rescue *Drosophila* mutant phenotypes [23–26].

DNA methylation is the process through which a methyl group is added to nucleotides in the DNA sequence. The most frequent target of DNA methylation in animals is cytosine bases present in CpG dinucleotides [27], although non-CpG methylation also occurs [28, 29], and is quite common in plants and some insect species [30–33]. In most organisms that exhibit DNA methylation, *de novo* methyltransferases establish DNA methylation, while maintenance methyltransferases replicate pre-existing methylation patterns as the DNA is replicated. DNA methylation at promoter sequences is frequently associated with repression of gene expression; however, methylation-requiring enhancers, repressors, and protein-binding sequences are also important in epigenetic gene regulation. Evidence suggests that DNA methylation and histone modifications frequently exhibit epigenetic “cross-talk”, with DNA methylation guiding histone modifications, and histone modifications similarly influencing DNA methylation [34, 35]. These two epigenetic processes therefore often function in a mutually reinforcing epigenetic loop that ensures maintenance of a repressive chromatin state.

RNAi pathways involve the processing of large coding or noncoding RNAs into small RNAs. These small RNAs can modify gene expression post-transcriptionally, by degrading an mRNA transcript or inhibiting its translation, or transcriptionally, by mediating chromatin modifications that promote the formation of heterochromatin and thereby inhibit transcription [8]. The molecular mechanisms underlying RNAi-directed heterochromatin formation have been most thoroughly studied in yeast, where transcripts from heterochromatic regions of the genome were found to be processed into siRNAs, which then recruited histone methylation that contributed to heterochromatin formation [36]. Noncoding RNA transcripts may also orchestrate changes in chromatin structure directly, rather than through an RNAi pathway, by mediating protein recruitment, histone modifications, and DNA methylation at a target site [8, 37, 38]. Both transcriptional and post-transcriptional RNAi-mediated silencing has been observed in a wide range of eukaryotic organisms, and key components of the RNAi machinery are conserved in plants, yeast, and animals [9]. The diverse range of RNAi-mediated pathways and processes that have been reported throughout the eukaryotic kingdom are therefore likely based on an evolutionarily conserved silencing process that was present in ancient eukaryotes.

## 3. Epigenetic Inheritance

Epigenetic changes to DNA sequences must be stably transmitted through mitosis, to ensure that the appropriate set of genes are expressed or repressed during growth and development, and cellular replacement and repair. Loss of the “correct” epigenetic state of a gene can lead to aberrant gene expression and the development of many types of cancer and other diseases [39, 40]. In addition to being mitotically heritable, epigenetic states can also be meiotically heritable, via mechanisms that result in a silent epigenetic state being inherited from one generation into the next. Genomic imprinting and paramutation are two epigenetic processes that exhibit this phenomenon of *trans*-generational epigenetic silencing.

Genomic imprinting is a process in which an allele is marked based on the sex of the parent transmitting it. This epigenetic mark can lead to transcriptional repression of fully functional alleles based strictly on whether they were inherited through the male or female germline. Imprinting has been observed in a wide range of eukaryotic organisms, including plants [41, 42], insects [43, 44], *C. elegans *[45], zebrafish [46], and mammals [47, 48]. In the process of imprinting, an epigenetic mark is differentially established in the male and female germlines, the maternal and paternal epigenetic states are maintained during the development of the organism, and finally the epigenetic states are erased in the gametes so that the organism transmits the “correct” epigenetic state to its offspring, according to whether it is male or female.

Paramutation is another *trans*-generational epigenetic silencing process, in which alleles of the same gene exhibit different epigenetic states. However, in paramutation, the epigenetic status of an allele is not dependent on its parent of origin, but it is instead influenced by the epigenetic status of an allele present in *trans*. In the process of paramutation, the epigenetic state and expression level of one allele changes after it is combined with another allele in a heterozygous organism. The allele's new epigenetic state is meiotically stable, and so it is inherited and maintained in the next generation [49].

Cross-species transgenic organisms have provided tremendous insight into the evolutionary conservation of the epigenetic silencing mechanisms underlying imprinting. Similarly, I have recently used transgenic *Drosophila* to examine the epigenetic mechanisms underlying maize paramutation. In order to assess whether this approach can successfully be used to examine epigenetic processes from other species, such as non-model organisms, this paper will summarize and examine the mechanistic and evolutionary insights gained from cross-species transgenic experiments studying genomic imprinting and paramutation.

## 4. Imprinting at the Mammalian *H19/Igf2* Locus

**Figure 1:**
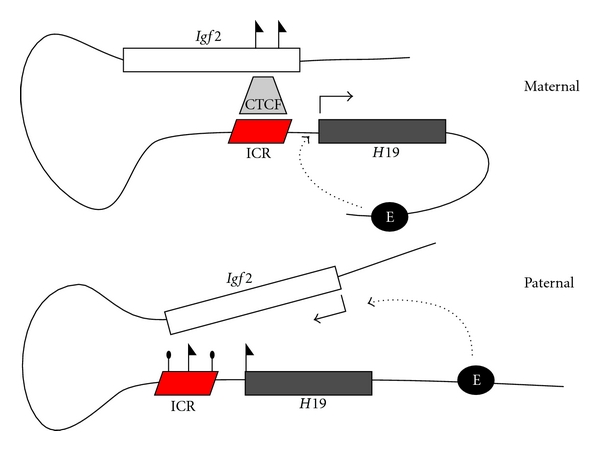
Imprinting at the *Igf2* and *H19* locus. On the maternally inherited allele, CTCF binds to the unmethylated ICR (red parallelogram) and the downstream enhancers (black circle) drive expression of *H19*. On the paternally inherited allele, the ICR is methylated (black lollipops), which prevents binding of the insulator protein CTCF, and enables the downstream enhancers to stimulate expression of *Igf2*. The maternal *Igf2* gene exhibits repressive histone marks (black flags), while these marks are found on the paternal ICR and *H19* gene.

In mammals, imprinted genes are often found in clusters that contain two or more imprinted genes, a shared imprint control region (ICR) or regions, and several gene-specific regulatory elements, all of which work together to establish and/or maintain the appropriate imprinted expression of the genes in the cluster. One of the best characterized examples of mammalian imprinting is that of *Insulin-like growth factor 2* (*Igf2*) and *H19*. *Igf2 *is expressed from the paternal allele only and is located approximately 90 kb from the noncoding *H19* transcript, which is expressed from the maternal allele only ([50, 51], Figure 1). A shared ICR that is required for imprinting of both genes is located approximately 2 kb upstream of the *H19* transcription start site [52]. In addition to the ICR, several tissue-specific enhancers 10–120 Kb downstream of the *H19* gene [53–56], several differentially methylated regions (DMRs) near *Igf2* [57–60], and a central A6-A4 DNAse hypersensitive region [61–63], are also important in establishing the correct expression profiles of these two imprinted genes.

Imprinting of *H19* and *Igf2 *require CCCTC-binding factor (CTCF), an enhancer-blocking insulator protein that is conserved from *Drosophila* to humans [64] and is similarly required for imprinting in *Drosophila *[65]. The *H19*/*Igf2* ICR contains several binding sites for CTCF which can only bind when these sites are unmethylated [66, 67]. Further, the ICR exhibits differential methylation in male and female gametes, with methylation detected in sperm but not oocytes [68]. Thus, CTCF is able to bind to the maternally inherited unmethylated ICR, but not the paternally inherited methylated ICR. The binding of CTCF to the maternal ICR blocks the downstream enhancers from activating *Igf2*, and instead the enhancers mediate expression of *H19*. Conversely, in the absence of CTCF on the methylated ICR of the paternal allele, the downstream enhancers activate expression of *Igf2* ([66, 67], Figure 1). Differential methylation of the ICR in the gametes therefore mediates differential binding of CTCF, and results in *H19 *expression from the maternally inherited chromosome only and *Igf2 *expression from the paternally inherited chromosome only.

Several differentially methylated regions (DMRs) also exist in the *Igf2* gene region. In mice, DMR0 is hypermethylated on the maternal allele in the placenta and encompasses the promoter of a placental-specific transcript [57]. DMR1 is a methylation-sensitive mesodermal repressor that is hypermethylated on the paternal allele and is required in the unmethylated state to mediate repression of the maternal *Igf2 *allele in mesoderm tissues [58, 60]. Conversely, DMR2 is a methylation-dependent *Igf2 *enhancer that is hypermethylated on the paternal allele and is important for stimulating high levels of paternal *Igf2* expression [59].

The process of *Igf2*/*H19* imprinting also involves the formation of higher-order chromatin structures. On the paternal chromosome, chromosome conformation capture (3C) has demonstrated interactions between the ICR and DMR2, as well as interactions between the *Igf2* promoter and downstream enhancers [69–71]. Conversely, the maternal chromosome exhibits chromatin interactions between the ICR and the *Igf2* promoter region, including DMR1, and between the downstream enhancers and the *H19* promoter [69–72]. In addition to binding at the ICR, CTCF binding is also detected at *Igf2 *at DMR1 and the two major *Igf2* promoters, P2 and P3 [69, 72]. Disruption of CTCF binding to the ICR also eliminates CTCF binding at DMR1, suggesting that the long range interactions between the ICR and *Igf2* gene region recruit CTCF to *Igf2*. CTCF binding at the maternal DMRs appears to protect these regions from acquiring the paternal-specific methylation pattern [69].

Histone modifications are also important in determining the correct patterns of *H19* and *Igf2* expression. The maternal *Igf2* allele is enriched for several repressive marks, including methylation at Lysine 9 of Histone H3 (H3K9), methylation at Lysine 27 of Histone H3 (H3K27), and the heterochromatic histone variant macroH2A1 [72–74]. H3K27 methylation is mediated by the highly conserved Polycomb repressive complex 2 (PRC2), which contains Suz12, a protein that can directly interact with CTCF [72]. Both H3K27 and Suz12 are required to maintain maternal *Igf2* repression [72], suggesting that the CTCF-mediated maternal chromatin loop represses *Igf2* by recruiting PRC2 to catalyze H3K27 methylation and maintain *Igf2* repression [72]. On the paternal chromosome, repressive histone modifications are found at the ICR and *H19* gene [73, 74]. Similarly, and consistent with their expression profiles, the paternal *Igf2* gene region is enriched for activating histone marks, such as histone acetylation and Histone H3 lysine 4 (H3K4) methylation, while these marks are predominant at the ICR and *H19* gene region of the maternal chromosome [73, 74].

CTCF is a master regulator of *H19* and *Igf2* imprinting, and elimination of CTCF binding to the maternal ICR causes the chromosome to adopt both the paternal pattern of histone modifications [73] and chromatin interactions [69]. Overall, the process of *Igf2* and *H19* imprinting is complex and requires an interplay between many underlying epigenetic mechanisms, including DNA methylation, histone modifications, higher-order chromatin structures, and chromatin binding proteins. Despite this complexity, cross-species transgenic experiments have provided many insights into the evolutionary conservation of genomic imprinting. 

## 5. *H19/Igf2* Transgenic Experiments

### 5.1. Human and Mouse → Drosophila

Epigenetic effects on gene expression, such as position effect variegation, telomeric position effect, *trans*-inactivation, and transvection, have been extensively studied in *Drosophila melanogaster* [75]. Given the evolutionary conservation of many epigenetic proteins and core epigenetic silencing mechanisms, transgenic *Drosophila* have also proven an invaluable tool for analysing epigenetic control sequences from other species. Early cross-species transgenic experiments examining *H19*/*Igf2* genomic imprinting in *Drosophila* provided insight into both the nature of the imprint control region and the evolutionary conservation of the mechanisms underlying genomic imprinting. In fact, a distinct silencer element contained within the mouse *H19 *ICR was discovered in transgenic *Drosophila *[76] prior to its identification at the endogenous mouse locus [77]. In this experiment, a 3.8 kb fragment of the *H19* upstream region, including most of the ICR, was found to silence both *lacZ* and mini-*white* reporter genes in transgenic *Drosophila. *Transgenic deletion constructs were able to further delineate the silencer element to a 1.2 kb region that includes approximately 900 bp of the 2 kb UTR. Importantly, subsequent experiments showed that targeted deletion of only this 1.2 kb silencer element in mice, while leaving the remainder of the UTR and surrounding region intact, caused a loss of *H19* silencing following paternal transmission but did not affect paternal *Igf2* expression, differential methylation of the UTR, or expression of *H19 *and *Igf2 *following maternal transmission [77]. Thus the mouse *H19* silencer that was discovered in transgenic *Drosophila* appears to be evolutionarily conserved in its function, acting as an epigenetic silencer both at its native locus and in the distantly related transgenic flies.

Transgenic *Drosophila* experiments also identified a similar 1.5 kb silencer element at the 3′ end of the human *H19* ICR [78]. This region silenced a mini-*white* reporter gene in transgenic *Drosophila*, while additional regions from the human ICR did not. The silencing activity of this specific fragment was confirmed in transient transfection assays using a human embryonic kidney cell line [78]. Interestingly, despite the lack of sequence similarity between the human and mouse ICRs, and the failure of the human ICR to imprint in mice (described in the next section), both ICRs appear to contain an evolutionarily conserved silencer element that functions in transgenic *Drosophila*.

Further examination of the mouse *H19/Igf2 *ICR in transgenic *Drosophila *provided additional evidence that the epigenetic mechanisms underlying genomic imprinting are conserved [79]. This study used transgenic *Drosophila *containing the larger 3.8 kb *H19 *upstream region, which includes the full ICR, and found that the ICR is transcribed in the sense and antisense direction, from both the maternal and paternal alleles, both at the endogenous mouse locus and in transgenic *Drosophila* [79]. The ability to rapidly test many mutant strains and easily manipulate the transgenic *Drosophila *system proved extremely useful in this study and provided further insight into the possible function of the ICR transcription. Mutations in several RNAi genes, including *piwi*, *aubergine*, *dicer-2*, *r2d2*, and *spindle-E*, failed to relieve reporter gene silencing in *Drosophila *[79]. Furthermore, despite the bidirectional transcription of the ICR, no siRNAs from the ICR could be detected in transgenic *Drosophila*. In fact, artificially producing *H19* ICR siRNAs by expressing a fragment of the ICR as an inverted repeat resulted in a significant reduction in the ICR transcripts and a loss of reporter gene silencing. Thus the *H19* ICR transcripts appear to induce gene silencing in an RNAi-independent manner [79]. At the endogenous mouse locus, the ICR transcripts may be involved in forming a repressive chromatin structure that contributes to paternal *H19* repression. Similar cases of a noncoding RNA transcript mediating the formation of a repressive chromatin structure have been observed at the imprinted *Cdkn1c-Kcnq1* domain [80], pericentric heterochromatin [81], and a ribosomal gene cluster [82]. Potentially, the repressive effect of the *H19* ICR transcripts on the maternal allele may be prevented or blocked by CTCF binding to this region, or the transcripts may serve a different functional role that has not yet been elucidated [79].

Similar transgenic experiments have shown that the central A6-A4 region also functions as a silencer in *Drosophila*. The placement of this region adjacent to mini-*white* and *lacZ* reporter genes in transgenic *Drosophila *resulted in overall silencing of both reporter genes and occasional eye pigment variegation, which is indicative of the formation of a repressive chromatin structure [83]. Silencing from the A6-A4 region in *Drosophila *may be consistent with the observation that this region includes a tissue-specific repressor in mice [62]. Again, transgenic *Drosophila *mutational analysis provided insight into the potential mechanism of repression. Two Polycomb group genes, *Enhancer of Zeste (E(z)) *and *Posterior Sex Combs (Psc), *were found to be important for reporter gene silencing and were observed to bind to the transgene integration site [83]. E(z) and Psc are highly conserved proteins that are involved in chromatin remodelling and the formation of repressive chromatin states [18]. The repressor activity of the A6-A4 region in mice may therefore require the mouse homologues of these proteins to mediate the formation of a condensed chromatin domain, although this has not yet been confirmed endogenously.

Overall, these transgenic results suggest that genomic imprinting in mammals may use evolutionary conserved silencing mechanisms. Silencers contained within the *H19/Igf2* mammalian imprint control regions are recognized and targeted for silencing in *Drosophila, *affecting the expression of nearby reporter genes. Bidirectional transcription of the ICR is also conserved between mice and *Drosophila*, and the transgenic *Drosophila *system was able to provide significant inscight into the potential role of these transcripts in mouse *H19/Igf2* imprinting. The silencing mediated by the upstream A6-A4 conserved region in *Drosophila *further suggests that the mechanisms governing epigenetic modifications at other sites important for imprinted expression are also conserved between mammals and flies, indicating tremendous evolutionary conservation of the mechanisms underlying this complex epigenetic process.

### 5.2. Human → Mocuse

The general mechanism of imprinting at the *Igf2*/*H19* locus shares many similarities between mice and humans, including the chromosomal arrangement of the two genes and the position of the ICR, the pattern of methylation and gene expression, and the binding of CTCF to the ICR. However, with the exception of the CTCF binding sites, the sequences of the mouse and human ICRs are significantly different [66, 67, 84], suggesting that some aspects of the mechanisms underlying imprinting might have diverged in the mammalian lineage.

Transgenic experiments confirm that there may be divergence in some of the mechanisms governing imprinted expression of *H19* between mice and humans. Mice containing a 100 kb human *H19* transgene failed to imprint the human *H19* gene [85], despite containing significantly more flanking sequence than a 15.7 kb mouse transgene that successfully imprinted in mice when present in a single transgene copy [2]. Interestingly, in all lines with more than a single copy of the 100 kb human *H19* transgene, methylation of the ICR was detected in sperm but not oocytes, with the level of methylation increasing as the number of transgene copies increased. Paternal methylation of the ICR began to decrease early in embryonic development and was undetectable in the somatic tissues of transgenic mice. *H19* was expressed equally after both paternal and maternal inheritance in all multi-copy lines. However, a single copy line showed a complete absence of both methylation and *H19* expression [85].

The requirement for multiple transgene copies to establish methylation at the ICR in the paternal germline may suggest that imprint signals in the human transgene are only weakly recognized in the mouse, and thus multiple copies are necessary to accumulate a strong enough signal for transgene methylation. Since methylation at the ICR of the human transgene is lost in the embryo, it is possible that the imprint is not established correctly or completely in the paternal germline, or is not recognized and maintained by the mouse machinery in the early embryo. While the presence of multiple transgene copies may trigger methylation that is unrelated to the ICR and imprinting machinery, as has been documented for other tandem repeats of transgenes [86, 87], this would not explain why the observed methylation was only acquired in the paternal germline, consistent with its epigenetic status in mice.

It would be intriguing to know whether the human ICR exhibits silencing activity in mice, despite failing to imprint or acquire methylation at the ICR region. Potentially, the human ICR and *H19* gene may acquire repressive histone modifications that lead to *H19* repression following both maternal and paternal inheritance. If the human ICR functions as a silencer in mice, this would be consistent with the lack of *H19* expression in the single-copy line, and with the transgenic *Drosophila* experiments described previously. In the case of the multi-copy lines, the silencing ability of the ICR may decrease as the copy number increases, resulting in equivalent *H19* expression following both maternal and paternal inheritance.

## 6. Transgenic Insights: Genomic Imprinting

The transgenic experiments described here indicate that several core epigenetic mechanisms underlying mammalian imprinting are highly conserved. This is perhaps not surprising given that plants, mammals, and insects utilize many of the same mechanisms to establish and maintain imprinted expression. These mechanisms include DNA methylation, histone modifications, changes in higher-order chromatin structure, and noncoding RNA and RNA interference, all of which are frequently interrelated and mutually reinforcing. Histone modifications have been observed to play an essential role in plant, insect, and mammalian imprinting and can result in parent of origin-specific higher-order chromatin structures or modifications that contribute to the imprinting of genes, gene clusters, or chromosomes [72, 88–91]. A homologous Polycomb complex participates in both plant and mammalian imprinting, further emphasizing their relatedness [72, 92, 93].

The fact that the human and mouse *H19/Igf2* imprint control regions function as silencers but do not confer imprinting of marker genes in *Drosophila* may indicate that a silenced epigenetic state is the default at these imprinted loci. In support of this, a human imprinting centre from the Prader-Willi/Angelman syndrome region was also found to function as a silencer in *Drosophila* [94]. In these experiments, a 740 bp sequence from the imprint centre was found to be sufficient for silencing of reporter genes, while other nonspecific DNA fragments exerted no effect [94].

The apparent divergence of *H19/Igf2* imprinting between mice and humans, despite the conservation of the underlying silencing mechanisms between mammals and *Drosophila*, may again indicate that the silent epigenetic state is the default. While silencing may use core widespread epigenetic mechanisms, such as histone modifications, DNA methylation, and higher-order chromatin modifications, imprinting may be a more divergent species-specific modification of these processes in the gametes. That is, the parent-of-origin patterns of gene expression observed in genomic imprinting may be accomplished by simply using conserved core epigenetic mechanisms differently in the maternal and paternal germ-line. However, it is important to note that the potential divergence between the human and mouse *H19* ICRs is not indicative that all imprinting processes at all imprinted loci have diverged between the two lineages. For example, transgenic mice containing sequence from the human Prader-Willi/Angelman syndrome domain successfully imprint the transgene [95–97], and subsequent experiments have found several *cis*-acting elements and protein binding complexes that function at the transgenic locus [98]. Similarly, a differentially methylated region near two paternally expressed human genes, *HYMAI* and *PLAG1*, was concluded to be an imprint control region following transgenic mouse experiments in which it successfully acquired differential methylation and conferred imprinting of an *eGFP* reporter gene [99]. It is therefore possible that including additional distant sequence, or modifying the sequence contained within the *H19 *transgene (e.g., by decreasing the distance between regulatory elements), could result in successful imprinting of the human *H19/Igf2* imprint control region in mice.

## 7. Paramutation at the Maize *B1* Locus

**Figure 2:**
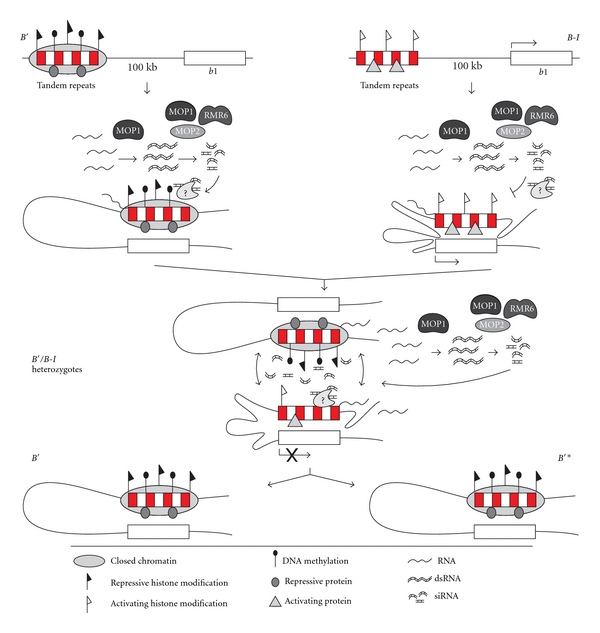
Paramutation at the maize *b1* locus. The two alleles that participate in paramutation at the *b1* locus are identical in sequence and contain an identical control region consisting of seven tandem repeats (red and white boxes). However, the *B-I* allele is highly transcribed while the *B′* allele is not. The two alleles exhibit epigenetic differences in chromatin structure, histone modifications, and DNA methylation and may be associated with distinct proteins that maintain these epigenetic states. The tandem repeats are bidirectionally transcribed in both *B-I* and *B′* plants, producing repeat RNA that then forms dsRNA and is processed into siRNAs. The proteins MOP1, RMR6, and MOP2 are important for the production and amplification of the dsRNA and siRNAs. The siRNAs are hypothesized to direct chromatin modifications at the tandem repeats via mechanisms and proteins that are currently unknown, but this process is blocked at the *B-I* allele, potentially by the active chromatin state, bound proteins, or nuclear environment. Paramutation occurs in heterozygous plants, when the highly transcribed *B-I* allele is “paramutated”, or converted, to the silenced *B′* state. siRNAs produced from the tandem repeats are hypothesized to mediate *trans*-interactions or communication between the alleles, as well as direct the establishment of a closed chromatin structure at the *B-I* tandem repeats. The conversion of *B-I* to a silenced epigenetic state is meiotically stable, and in the next generation all progeny will inherit a silenced *B′* allele. The newly paramutated allele is termed B′*.

The maize *b1* locus provides one of the best characterized examples of paramutation. The *b1* gene encodes a transcription factor that regulates expression of genes required for the synthesis of purple anthocyanin pigments [100]. Two alleles at the *b1* locus participate in paramutation: the highly transcribed *B-I *allele, and the weakly transcribed *B′* allele. Paramutation occurs when a *B-I *allele is combined with a *B′* allele in heterozygous plants, and results in epigenetic silencing of the normally highly expressed *B-I *allele ([101, 102], Figure 2).

Paramutation at the *b1 *locus requires a control region that is located 100 kb upstream of the *b1* transcription start site and consists of a 6 kb sequence containing seven tandem repeats of an 853 bp sequence [103]. Despite containing identical DNA sequences, the *B′* tandem repeats exhibit a closed chromatin structure, repressive histone modifications, and higher levels of DNA methylation, whereas the *B-I *tandem repeats have an open chromatin structure and histone H3 acetylation, an activating histone modification [103, 104].

Higher-order chromatin structure may also play a role in determining the epigenetic status of the repeats and the transcriptional status of *B-I *and* B′* [105]. Chromosome conformation capture (3C) has demonstrated that the high-expressing *B-I* allele exhibits a higher frequency of chromatin interactions than *B′*, involving the transcription start site, the tandem repeats, and several additional upstream regulatory regions, suggesting the formation of a complex multi-loop structure that facilitates *b1* expression. In contrast, the weakly expressed *B′* allele exhibits less frequent interactions involving only the transcription start site and the tandem repeats, suggesting the formation of a less stable single-loop structure [105].

Paramutation at the *b1* locus requires several proteins, including *mediator of paramutation 1*, or *mop1* [106], which encodes an RNA dependent RNA polymerase [107], *mediator of paramutation 2* (*mop2*, also known as *rmr7*), which encodes the second largest subunit of both RNA polymerases IV and V [108], and *required to maintain repression 6* (*rmr6*) [109], which encodes the largest subunit of RNA polymerase IV [110]. In *Arabidopsis*, these RNA polymerases participate in the production of siRNAs and noncoding RNAs, transcriptional gene silencing, silencing of transposons and repetitive DNA, RNA-directed DNA methylation, and heterochromatin formation [111–116]. In addition, a protein termed CXC domain *b1*-repeat binding protein, or CBBP, has been shown to bind to the *b1* tandem repeats and appears to be involved in establishing, rather than maintaining, the silenced epigenetic state [117].

The maize *b1* tandem repeats are transcribed from both strands, with a similar level of transcription in *B-I *and* B′* plants, as well as in plants with a neutral allele that contains only a single copy of the repeat [107]. 24-nt siRNAs from the tandem repeats have been detected in plants with *B-I*, *B′*, and the single-repeat allele, but are reduced in the presence of a *mop1* or *mop2 *mutation [108, 118]. It is thus likely that the bidirectional transcription of the repeats produces double stranded RNA (dsRNA) and that MOP1 and MOP2 are required to produce significant levels of siRNAs from the dsRNA molecules.

The current model for paramutation at the *b1* locus is that RNA-mediated communication between the *B-I *and* B′* alleles establishes the chromatin states of the control regions, which thereby determines the level of *b1* transcription ([118, 119], Figure 2). The open chromatin structure of the *B-I* tandem repeats, and the multi-chromatin loops that are formed at this allele, may promote *b1* transcription, whereas the closed chromatin structure of the *B′* tandem repeats, and single chromatin loop, may inhibit or prevent *b1* transcription. Importantly, however, the presence of siRNAs in the nonparamutating single-repeat allele suggests that the siRNAs alone are not sufficient to induce paramutation. The number of tandem repeats is also important and may mediate or stabilize pairing-interactions between alleles, potentially via increased accumulation of proteins or chromatin modifications. In addition to RNA-mediated communication, interactions between the DNA sequences, proteins bound to the DNA, or the formation of higher-order protein complexes may also play a role in paramutation by bringing the two alleles together physically, or localizing them to a particular nuclear compartment where silencing and a heritable chromatin state can be established by the siRNAs [119].

Given that the highly expressed *B-I *allele contains seven tandem repeats that are transcribed and produce siRNAs, there is necessarily an additional mechanism that normally prevents silencing at this allele. The active chromatin structure of the repeats, or specific proteins that bind to the active chromatin structure, may inhibit the formation of the silenced epigenetic state, or the allele may be localized to a different nuclear environment that inhibits silencing [118, 119]. Alternatively, pre-existing repressive modifications at the *B'* allele may make it susceptible to further siRNA-directed modifications [104]. This could be similar to the mechanism at the *Arabidopsis FWA *locus, where siRNAs direct DNA methylation at the tandem repeats of silenced alleles with pre-existing methylation, but not at active epialleles [120].

## 8. Transgenic Experiments

### 8.1. Maize → Drosophila

**Figure 3:**
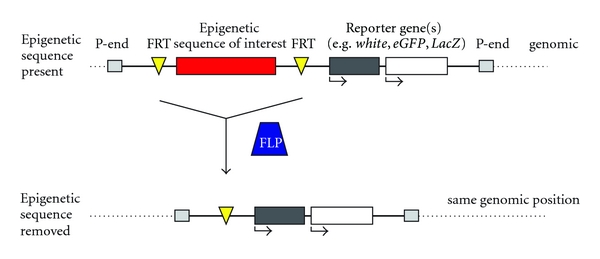
An FRT-FLP mediated approach that can be used to analyze any epigenetic sequence of interest from any organism, in *Drosophila*. The epigenetic sequence of interest is placed between two FRT sites, with one of more reporter genes positioned adjacent to the sequence of interest, but outside of the FRT sites. Following incorporation into the genome, the transgenic line can be crossed to a source of FLP recombinase, which excises FRT-flanked DNA sequences and thereby mediates removal of the test epigenetic sequence. This approach produces transgenic *Drosophila* with reporter genes at the same genomic position, but with or without the epigenetic sequence of interest present, thereby controlling for nonspecific genomic and position effects on reporter gene expression.

In order to analyze the conservation of epigenetic mechanisms underlying maize *b1 *paramutation, I generated transgenic *Drosophila* carrying the seven maize *b1* tandem repeats adjacent to the *Drosophila white* reporter gene [121]. In this transgenic system, the *b1* repeats are located between two Flipase Recombinase Target (FRT) sequences. This experimental design allows for the removal of the repeats by crossing to a source of Flipase recombinase (FLP), which mediates site-specific removal of FRT-flanked sequences, thereby enabling analysis of reporter gene expression from transgenes with and without the *b1* repeats, at identical chromosomal positions. A similar type of construct could theoretically be used to examine the effect of any epigenetic sequence of interest, from any organism, on reporter genes in *Drosophila* (Figure 3).

In all eleven transgenic *Drosophila *lines examined, the maize* b1* repeats functioned as a silencer, with a visible increase in *white *expression observed following repeat removal [121]. Silencing strength increased as the number of tandem repeats increased; however, silencing was also observed with a single copy of the repeat sequence, indicating that the observed silencing of *white *is not due to nonspecific silencing of a tandem repeat array, and further suggesting that each 853 bp tandem repeat contains evolutionarily conserved silencing sequences that are recognized and targeted for silencing in transgenic *Drosophila*. Importantly, this is in contrast to other experiments in which no effect on *white* expression was observed for *Drosophila* containing tandem repeats from mosquito subtelomeric heterochromatin [122], and no change in *white* expression was observed following FLP-mediated removal of adjacent repetitive sequence from the *Drosophila 1360* transposable element [123]. Thus, the maize repeats appear to specifically recruit epigenetic silencing in transgenic *Drosophila*.

Evidence of *trans-*silencing was also observed in transgenic *Drosophila*, as several transgenes exhibited reduced expression when homozygous. *Trans*-silencing was only observed when transgenes at the same genomic position were combined, suggesting that it requires direct pairing of the homologous transgenes. Further, the *b1 *control region produces bidirectional transcripts in the transgenic *Drosophila* system [121], as in the endogenous maize system. Interestingly, despite the role of the *b1 *tandem repeats in both mediating paramutation and acting as an enhancer that drives high expression of the *B-I* allele [103], *cis* activation of the *white *transgene by the tandem repeats was not observed in *Drosophila *[121]. This may indicate that the silent epigenetic conformation of the *b1 *tandem repeats is the default epigenetic state. This agrees with the observation that the highly expressed *B-I *allele will spontaneously convert to the silenced* B′* state at a frequency of 1–10% [101, 102], whereas the reverse, conversion of the silenced *B′* state to the active *B-I* state, has never been observed [101, 119]. In maize, it is likely the case that the mechanism used to ensure high levels of transcription of the *B-I *allele occasionally fails, causing the allele to adopt the default silenced epigenetic state [118]. Similarly, while the default silenced epigenetic state is readily adopted in transgenic *Drosophila*, the mechanisms required for the tandem repeats to act as an enhancer are likely absent, unstable, or fail. Alternatively, this may be explained by the fact that the *b1 *tandem repeats are only ~500 bp from the *white *reporter gene in transgenic *Drosophila*, whereas in maize, the *b1 *gene is ~100 kb from the tandem repeats. This greater distance may be required for the formation of the higher order chromatin loops, which involve additional upstream sequences, and may facilitate the formation of the active epigenetic state [105]. As paramutation necessarily requires both active and silent epialleles, conservation of the full paramutation process could not be assessed in the transgenic *Drosophila* system.

## 9. Transgenic Insights: Paramutation

The transgenic *Drosophila *results suggest that paramutation is likely based on conserved epigenetic silencing mechanisms, as silencing was observed in all transgenic lines containing the *b1* tandem repeat sequence [121]. Similarly, it has recently been proposed that paramutation employs widespread RNA-based mechanisms that direct epigenetic modifications at target sites, and is simply an extreme manifestation, or aberration, of these processes [124, 125]. The transgenic results agree with these assertions, as evidence of silencing, *trans-*interactions, and bidirectional transcription, are all observed when the *b1* control region is moved to a new species. Further, the extensive evolutionary distance between maize, an angiosperm plant, and *Drosophila, *a dipteran insect, provides support for the hypothesis that core epigenetic mechanisms are conserved throughout the eukaryotic kingdom, and seemingly unique epigenetic phenomena function by exploiting these core mechanisms.

## 10. Conclusions

Transgenic organisms have proven to be an extremely valuable tool for studying a wide range of epigenetic processes. While the traditional transgenic method of moving an epigenetic control region to a novel chromosomal position in the same organism has been widely used, an alternate, but underutilized, method involves using transgenic organisms to examine epigenetically-regulated regions from a different species. The examples described here clearly demonstrate that significant insight into both the molecular mechanisms and the evolutionary conservation of these mechanisms may be obtained by examining epigenetic control regions from one species in another. As non-model organisms are traditionally difficult to work with in a laboratory environment, this approach should prove especially useful in future non-model organism epigenetic studies.

*Drosophila,* in particular, have long been a valuable resource for a wide range of genetic and epigenetic studies. The conservation of core epigenetic mechanisms enhances the utility of *Drosophila *in transgenic epigenetic studies, as the transgenic system can be used to advance the understanding of the molecular mechanisms and proteins that function at the endogenous locus. Transgenic *Drosophila* also provide a unique opportunity to study the molecular mechanisms of epigenetic processes from other species in more detail. A tremendous number of *Drosophila* mutant strains are readily available and can be tested with relative speed and ease, compared to mutational testing in other species.

While an epigenetic control region does not always successfully confer an epigenetic process in full when transferred to a novel organism, it frequently causes an observable epigenetic effect on adjacent marker genes. For example, imprint control regions from mice and humans cause gene silencing, but not imprinting, in *Drosophila*, and a paramutation control region from maize causes silencing and pairing-sensitive *trans-*silencing, but not paramutation, in *Drosophila*. Despite not fully recreating the endogenous epigenetic effect, these experiments nonetheless allow for dissection of the underlying conserved epigenetic processes from the species-specific modulations of these processes. Indeed, such experiments allowed for the discovery of a silencer at the mouse *H19*/*Igf2* imprint control region in transgenic *Drosophila*, prior to its discovery in mice. Even the apparent failure of a human imprint control region to function in transgenic mice is of interest, as it indicates that additional sequences may be required for successful imprinting, or that species-specific modification of a finely tuned epigenetic mechanism may have led to rapid evolution of imprinting control between the two mammalian species.

It is important to note that this methodology is not without its caveats. It is possible that an epigenetic control region may recruit different proteins or modifications in a transgenic organism than at the endogenous locus, or it may fail to recruit any epigenetic modifications in the transgenic environment, despite conferring epigenetic effects in the original organism. Species-specific unique proteins may function at the endogenous locus, preventing these interactions from being detected in the transgenic organism, or similarly, species-specific proteins may be recruited to the transgenic locus, leading to false conclusions regarding the processes involved in the original species. Importantly, the proteins or modifications recruited to the transgenic sequence may be influenced by the larger genomic environment, including the proximity of nearby heterochromatin domains and the identity of neighbouring regulatory sequences and genes, necessitating examination of several transgenic lines with different transgene insertion sites. It is therefore best, whenever possible, to use these transgenic experiments to provide insight and guide new studies in the original species that could lead toward confirmation of the results.

In all, the transgenic experiments described here suggest that seemingly unique epigenetic processes, such as genomic imprinting and paramutation, function via exploitation of conserved epigenetic mechanisms. Given the silencing observed from imprinting and paramutation control regions in transgenic *Drosophila*, it appears likely that the silencing mechanisms underlying these processes are core mechanisms that are highly conserved from one species to another, while the unique patterns of gene expression observed at the endogenous loci are due to species-specific modulations of these mechanisms. As many epigenetic proteins and processes are highly conserved, and transgenic model organisms have proven useful in analyzing epigenetic control regions from other species, this approach holds great promise for future non-model organism epigenetic studies.
